# MiR-126, IL-7, *CXCR1/2* receptors, inflammation and circulating endothelial progenitor cells: The study on targets for treatment pathways in a model of subclinical cardiovascular disease (type 1 diabetes mellitus)

**DOI:** 10.1186/s12967-021-02785-7

**Published:** 2021-04-16

**Authors:** David J. Coulson, Sherin Bakhashab, Jevi Septyani Latief, Jolanta U. Weaver

**Affiliations:** 1 Translational & Clinical Research Institute, Newcastle University, Newcastle Upon Tyne, NE2 4HH UK; 2 Biochemistry Department, Faculty of Science, King Abdulaziz University, P.O. Box 80218, Jeddah, Saudi Arabia; 3 Department of Diabetes, Queen Elizabeth Hospital, Gateshead, Newcastle Upon Tyne, NE9 6SH UK; 4 Vascular Biology and Medicine, Newcastle University, Newcastle Upon Tyne, NE2 4HH UK

**Keywords:** MiR-126-5p, *CXCR1*/*2*, IL-7, Inflammation, CVD, T1DM

## Abstract

**Background:**

Type 1 diabetes (T1DM) is associated with premature cardiovascular disease (CVD) and a pro-inflammatory state whilst the proangiogenic miR-126-3p/-5p may play a role in CVD. Animal studies established miR-126 to be pro-angiogenic. We hypothesised miR-126-3p/-5p are reduced in T1DM whilst pro-inflammatory cytokines are increased.

**Methods:**

29 well controlled, T1DM patients without CVD and 20 healthy controls (HCs) were studied. MiR-126-3p/-5p were assayed in plasma and peripheral blood mononuclear cells (PBMCs) whilst Chemokine C-X-C Receptor 1/2 (*CXCR1/2*) mRNA in PBMCs by real-time quantitative PCR. Cytokines were assayed by the Mesoscale Discovery. Ingenuity Pathway Analysis (IPA) was used to predict target genes, cellular functions and pathological states regulated by miR-126-3p/-5p. IPA generated both direct and indirect causations between different targets and analysed whether these effects would be inhibitory or stimulatory based on the published evidence.

**Results:**

T1DM patients had a relatively good diabetic control (HbA1c = 7.4 ± 0.7% or 57.3 ± 7.6 mmol/mol). Homeostatic cytokine IL-7, pro-inflammatory cytokines IL-8 and TNF-α, and vascular endothelial growth factor-C (VEGF-C) were increased in T1DM, versus HCs; *p* = 0.008, *p* = 0.003, *p* = 0.041 and *p* = 0.013 respectively. MiR-126-5p was significantly upregulated in PBMCs in T1DM versus HCs; *p* = 0.01, but not in plasma. MiR-126-3p was unchanged. *CXCR1/2* were elevated in T1DM versus HCs; *p* = 0.009 and *p* < 0.001 respectively. MiR-126-5p was positively correlated with *CXCR1/2*, and with HbA1c whilst negatively correlated with circulating endothelial progenitor cells (CD34^+^CD133^+^CD45^dim^) and fibronectin adhesion assay in a combined group of T1DM patients and HCs; *p* = 0.028 *p* = 0.049 *p* = 0.035 *p* = 0.047 and *p* = 0.004 respectively. IPA predicted miR-126-5p to be anti-inflammatory through the inhibition of chemokine C–C motif ligand 27, chymotrypsin-like elastase 2A and IL-7, whilst miR-126-3p had no direct anti-inflammatory effect. Simultaneously IPA predicted IL-7 as the most upstream cytokine target.

**Conclusions:**

T1DM without apparent CVD or diabetic complications is an inflammatory state characterised not only by raised pro-inflammatory cytokines but also by increased receptor *CXCR1/2* and miR-126-5p. MiR-126-5p upregulation may represent a compensatory response. Pro-miR-126-5p therapies or anti-IL-7 therapies may be a new option to reduce both inflammation and CVD risk in T1DM. Further research is required in a large prospective study in patients with T1DM.

## Background

Cardiovascular disease (CVD) remains the leading cause of mortality worldwide. An increased incidence of CVD is evident in type 1 diabetes mellitus (T1DM) and is the major contributor to early mortality in T1DM [1]. CVD is closely associated with inflammation; pro-inflammatory cytokines, drivers of CVD in humans [2]. While pro-inflammatory cytokines can serve as biomarkers in CVD [2] microRNAs (miRNAs) have also emerged as biomarkers in CVD including acute myocardial infarction, heart failure and coronary artery disease [3].

MiRNAs are small non-coding ~ 22 nucleotides long RNAs, which negatively regulate gene expression post-transcriptionally by binding to the 3′UTRs of mRNAs and blocking either translation or promoting mRNA degradation. MiRNAs can be detected in peripheral blood mononuclear Cells (PBMCs) and plasma [3]. Plasma miRNAs have extraordinary stability thought to be conferred through incorporation in micro-particles or association with RNA-binding proteins or lipoprotein complexes [3]. MiRNAs are tissue-specific and disease-specific, with dysregulation of miRNAs observed in disease states including CVD. The ease of assaying miRNAs in the blood in conjunction with tissue- and disease-specific expression, makes miRNAs ideal as potential biomarkers. The proangiogenic miR-126 isoforms have been extensively studied as biomarkers in CVD due to their dysregulated expression in CVD [3].

The miRNA precursor pre-miR-126 is processed to form two miR-126 mature isoform strands, miR-126-3p/-5p, both of which are abundant in endothelial cells [4, 5]. The miR-126 mature isoforms are demonstrated to play roles in angiogenesis and increasing vascular integrity in zebrafish [4]. Thus, the association of T1DM with CVD warrants the investigation of the proangiogenic miRNAs, miR-126-3p/-5p. We hypothesise that pro-inflammatory cytokines are upregulated in T1DM whereas the proangiogenic miRNAs, miR-126-3p/-5p, are downregulated.

## Methods

### The study

In our study 29 T1DM patients and 20 age- and sex-matched healthy controls (HCs), were recruited. Blood samples were collected for clinical and cytokine analysis. The study was approved by the NHS Health Research Authority, NRES Committee North East-Sunderland, UK (Research Ethics Committee Reference Number 12/NE/0044). All subjects provided informed consent and the study was performed in accordance with the Helsinki Declaration.

### Clinical analysis and vascular health

In T1DM patients and HCs clinical characteristics were acquisitioned as described previously [6]. Similarly, fibronectin adhesion assays and the quantification of the circulating endothelial progenitor cells markers CD34^+^CD133^+^CD45^dim^ were performed as described previously [6].

### Cytokine analysis

After an overnight fast IL-7, IL-8, TNF-α and vascular endothelial growth factor-C (VEGF-C) were assayed in plasma using K15050D V-PLEX Cytokine Panel 1 human kit, K15049D V-PLEX Proinflammatory Panel 1 human kit (Meso Scale Discovery, Rockville, MD, USA) according to the manufacturer’s instructions. Plates were read with MSD Sector Imager 2400, and data were analysed using the MSD Discovery Workbench v. 2.0 software.

### Extraction of miRNAs from plasma

Blood samples were centrifuged for 15 min at 500×*g.* The upper fraction containing platelet-rich plasma was collected and centrifuged for 5 min at 13,000×*g*. The clarified platelet-free plasma was collected and stored at − 80 °C for subsequent analysis. Samples were checked for haemolysis to ensure the samples were not contaminated with cellular miRNA. An aliquot of 200 μL per sample was transferred to a FluidX tube and 60 μl of Lysis solution BF containing 1 μg carrier-RNA per 60 μl Lysis Solution BF and RNA spike-in template mixture was added to the sample and mixed for 1 min and incubated for 7 min at room temperature, followed by addition of 20 μL Protein Precipitation solution BF. Total miRNA was extracted from plasma using miRCURY RNA isolation Kit—Biofluids; high-throughput bead-based protocol v.1 (Exiqon, Vedbaek, Denmark), optimized for serum/plasma by QIAGEN (Exiqon Services, Vedbaek, Denmark). The integrity of RNA samples was assessed using Agilent 2100 (Santa Clara, CA, USA) yielding high RNA Integrity Numbers (RIN) between 9.1 and 10.

### Extraction of miRNA and mRNA from PBMCs

After an overnight fast, peripheral blood was collected and PBMCs were isolated by Ficol separation. Cells were lysed with trizol lysis buffer and lysates were stored at − 80 °C for further analysis. Total RNA was extracted from PBMCs using the miRNEasy Kit (QIAGEN, Hilden, Germany). The integrity of RNA samples was assessed using Agilent 2100 yielding high RIN between 9.1 and 10.

### MiRNA and mRNA quantitative real-time PCR

MiRNAs were assayed by quantitative real-time PCR (qRT-PCR) in plasma and PBMCs with the miRCURY LNA RT Kit (QIAGEN, Hilden, Germany). 10 ng RNA was reverse transcribed, the resulting cDNA was diluted 100× and assayed in 10 μl PCR reactions in accordance with the protocol for miRCURY LNA miRNA PCR. Each miRNA was assayed once by qPCR on the miRNA Ready-to-Use PCR, Pick and Mix using miRCURY LNA SYBR Green master mix. Negative controls from the reverse transcription reaction were performed and profiled similarly to the samples. The amplification was performed in a LightCycler® 480 Real-Time PCR System (Roche, Basel, Switzerland). has-miR-126-3p (Cat number: YP00204227) and hsa-miR-126-5p (Cat number: YP00206010) were assayed using miRCURY LNA miRNA PCR Assays (Qiagen). Normalization was performed based on the average of the assays detected in all plasma samples using global mean normalization method.

For the present study, this included 7 assays:

hsa-miR-21-5p (TAGCTTATCAGACTGATGTTGA),

hsa-miR-320a (AAAAGCTGGGTTGAGAGGGCGA),

hsa-miR-23a-3p (ATCACATTGCCAGGGATTTCC),

has-miR-92a-3p (TATTGCACTTGTCCCGGCCTGT),

hsa-miR-223-3p (TGTCAGTTTGTCAAATACCCCA),

hsa-miR-126-3p (TCGTACCGTGAGTAATAATGCG),

hsa-miR-15a-5p (TAGCAGCACATAATGGTTTGTG).

Whereas, miRNAs from PBMCs were normalized to the average of 11 assays (hsa-miR-30e-3p, Cat number: YP00204410; hsa-miR-365a-3p, Cat number: YP00204622; hsa-miR-374b-5p, Cat number: YP00204608; hsa-miR-26b-3p, Cat number: YP00204117; hsa-miR-576-5p, Cat number: YP00206064; hsa-miR-425-3p, Cat number: YP00204038; hsa-miR-454-5p, Cat number: YP00204279; hsa-miR-769-5p, Cat number: YP00204270; hsa-miR-200c-3p, Cat number: YP00204482; hsa-miR-660-5p, Cat number: YP00205911; hsa-miR-331-3p, Cat number: YP00206046; Qiagen) detected in all samples.

Amplification curves were analysed with the Roche LC software to determine ΔCt values. ΔΔCt values were calculated with the Eq. 1 and fold change calculated with Eq. 2.

1 $$ \Delta \Delta Ct \, = \, \left( {\Delta Ct \, T1DM} \right) \, {-} \, \left( {\Delta Ct \, Healthy \, Controls} \right) $$

2 $$ Fold \, Change \, = \, 2^{|\Delta \Delta Ct|} \quad If \, \Delta \Delta Ct < \, 0,Fold \, Change \, = \, - (2^{|\Delta \Delta Ct|} )\, $$

Reverse transcription of 150 ng PBMC mRNAs was performed using QIAGEN RT ^2^ First Strand Kit (QIAGEN, Hilden, Germany), then cDNA was assayed using RT^2^ Profiler™ Custom Human PCR Array (QIAGEN, Hilden, Germany) containing *CXCR1* (Cat number: PPH01040F) and *CXCR2* (Cat number: PPH00608F). All Cq data was normalised to reference genes: actin-β (Cat number PPH00073G), lactate dehydrogenase A (Cat number: PPH02047H), hypoxanthine phosphoribosyltransferase 1 (Cat number: PPH01018C), ribosomal protein, large, P0 (Cat number: PPH21138F), β-2-microglobulin (Cat number: PPH01094E), and glyceraldehyde-3-phosphate dehydrogenase (Cat number: PPH00150F) yielding ΔCq. The gene expression fold change was calculated by the 2^ΔΔCt^ method in Eqs. 1 and 2.

Ingenuity pathway analysis (IPA) software version 9 (Ingenuity, Redwood City, CA, USA) supported the determination of cellular functions, pathways and genes regulated by miR-126-3p/-5p. IPA generated both direct and indirect causations between different targets and analysed whether these effects would be inhibitory or stimulatory based on the published evidence.

### Statistical analyses

All data is presented as mean ± SD, unless stated. Normality of the data was assessed, and unpaired *t*-tests or Mann Whitney U-tests were used accordingly to assess significant differences. Correlation analysis was performed using a two-tailed Pearson’s correlation coefficient. All statistical analyses were performed using IBM™ SPSS™ software version 25 (SPSS™ Inc., Armonk, NY, USA), at a significance level of *p* < 0.05.

## Results

### Patient characteristics

Characteristics of T1DM patients and HCs are provided in Table 1.

**Table 1:** Subjects’ clinical and metabolic characteristics

	HC (n = 20)	Type 1 diabetes (n = 29)	p-value
Age (years)	46.5 ± 11.7	47.2 ± 12.7	0.8
BMI (kg/m^2^)	26.0 ± 4.5	28.4 ± 6.7	0.3
Gender (M/F) n	9/11	14/15	–
HbA1c (mmol/mol)	35.1 ± 2.8	57.3 ± 7.6	< 0.001***
HbA1c (%)	5.4 ± 0.3	7.4 ± 0.7	< 0.001***
Glucose (mmol/L)	4.8 ± 1.0	9.7 ± 3.7	< 0.001***
CD34^+^ CD133^+^ per 100 lymphocytes	0.09 ± 0.03	0.02 ± 0.01	< 0.001***
Fibronectin adhesion assay per hpf	68.6 ± 34.5	33.9 ± 14.7	0.01*

Body mass index (BMI), male (M), female (F), high power field (hpf). *p < 0.05, ** *p* < 0.01, ***p < 0.001

### Cytokine profiles

Homeostatic cytokine IL-7, pro-inflammatory cytokines IL-8 and TNF-α, and the growth factor VEGF-C were elevated in T1DM patients (2.3 ± 0.6 pg/ml, 4.7 ± 1.3 pg/ml, 1.6 ± 0.2 pg/ml and 63.2 ± 20.3 pg/ml respectively) versus HCs (1.4 ± 0.6 pg/ml, 2.8 ± 0.5 pg/ml, 1.4 ± 0.2 pg/ml and 50.8 ± 48.2 pg/ml respectively); *p* = 0.008, *p* = 0.003, *p* = 0.041 and *p* = 0.013 respectively as reported by us recently [7]. In PBMCs *CXCR1* and *CXCR2* mRNA was significantly upregulated in T1DM versus HCs; as recently published by us [7]. The CXCR1:CXCR2 mRNA ratio was unchanged between T1DM and HCs.

### MiR-126 isoform expression

**Fig. 1:**
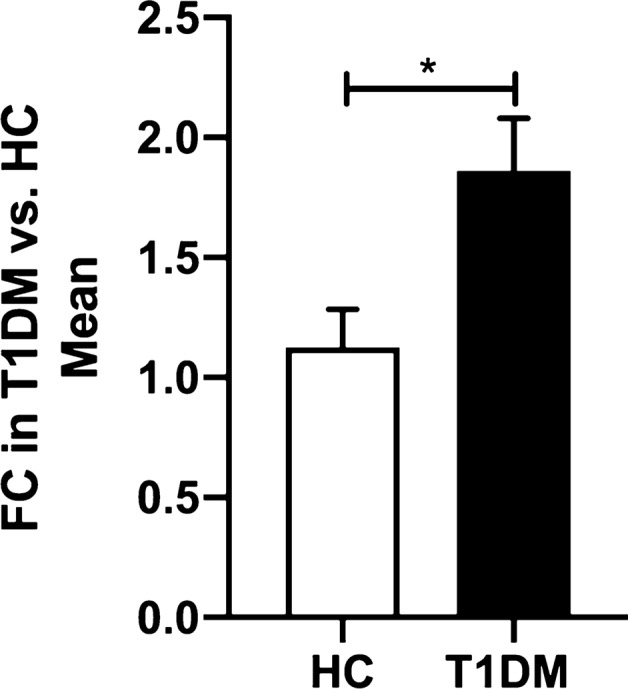
Comparison of miR-126-5p expression between HC and T1DM patients in PBMCs. Data are represented as mean ± SEM and analysed by unpaired *t*-tests. *p < 0.05

In PBMCs miR-126-5p was significantly upregulated in T1DM; fold change = 1.8 *p* = 0.01 versus HC but not in plasma whilst miR-126-3p was unchanged in both plasma and PBMCs (Fig. 1).

### Correlations in a combined group of T1DM patients and HCs

**Fig. 2:**
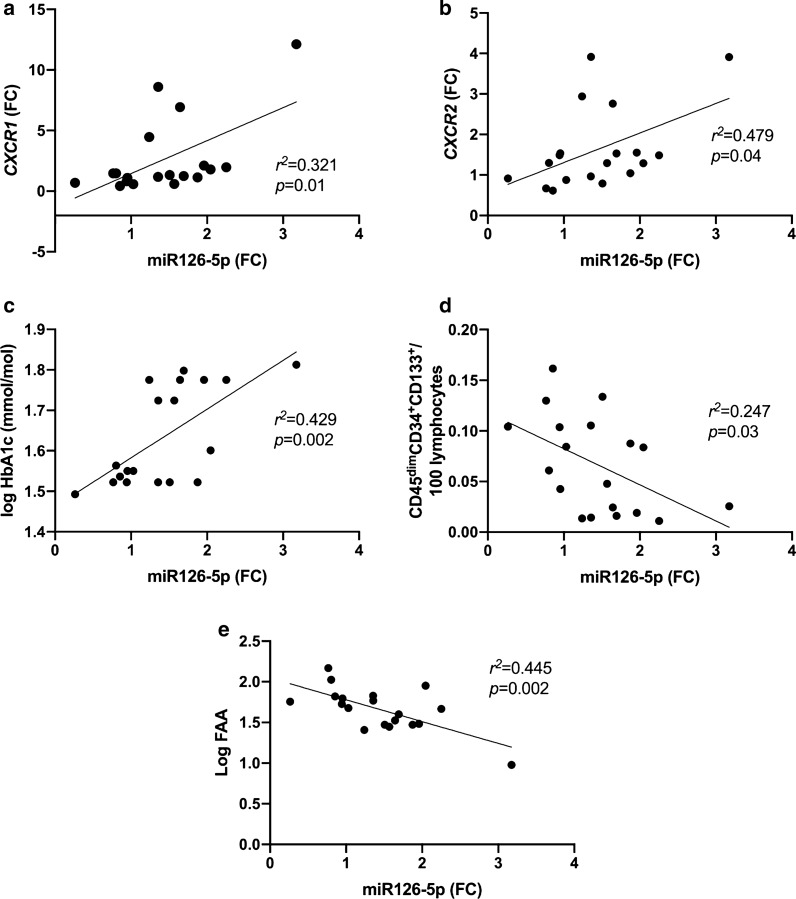
Correlation between miR-126-5p and pro-inflammatory receptors, HbA1c and vascular health in T1DM patients and healthy controls. **a**, **b** positive correlations of miR-126-5p with *CXCR1* and *CXCR2*; and **c** miR-126-5p with HbA1c; **d** negative correlation between miR-126-5p with CD45^dim^CD34^+^CD133^+^ per 100 lymphocytes; and **e** miR-126-5p with log FAA. FAA: fibronectin adhesion assay; FC: fold change

MiR-126-5p was positively correlated with *CXCR1* and *CXCR2* mRNA in the combined group; *r*^*2*^ = 0.321, *p* = 0.01 and *r*^*2*^ = 0.479, *p* < 0.05 respectively (Fig. 2a, b), but no significant correlation was detected with pro-inflammatory cytokines IL-8 and TNF- α, homeostatic cytokine IL-7 and VEGF-C. A positive correlation of miR-126-5p with HbA1c was observed in the combined group; *r*^*2*^ = 0.429, *p* = 0.002 (Fig. 2c). Whilst miR-126-5p was negatively correlated with CD45^dim^CD34^+^CD133^+^ cells per 100 lymphocytes and fibronectin adhesion assay counts (vascular health) in the combined group; *r*^*2*^ = 0.247, *p* = 0.03 and *r*^*2*^ = 0.445, *p* = 0.002 respectively (Fig. 2d, e).

### Receiver operating characteristic curve analysis for miR-126-5p

**Fig. 3:**
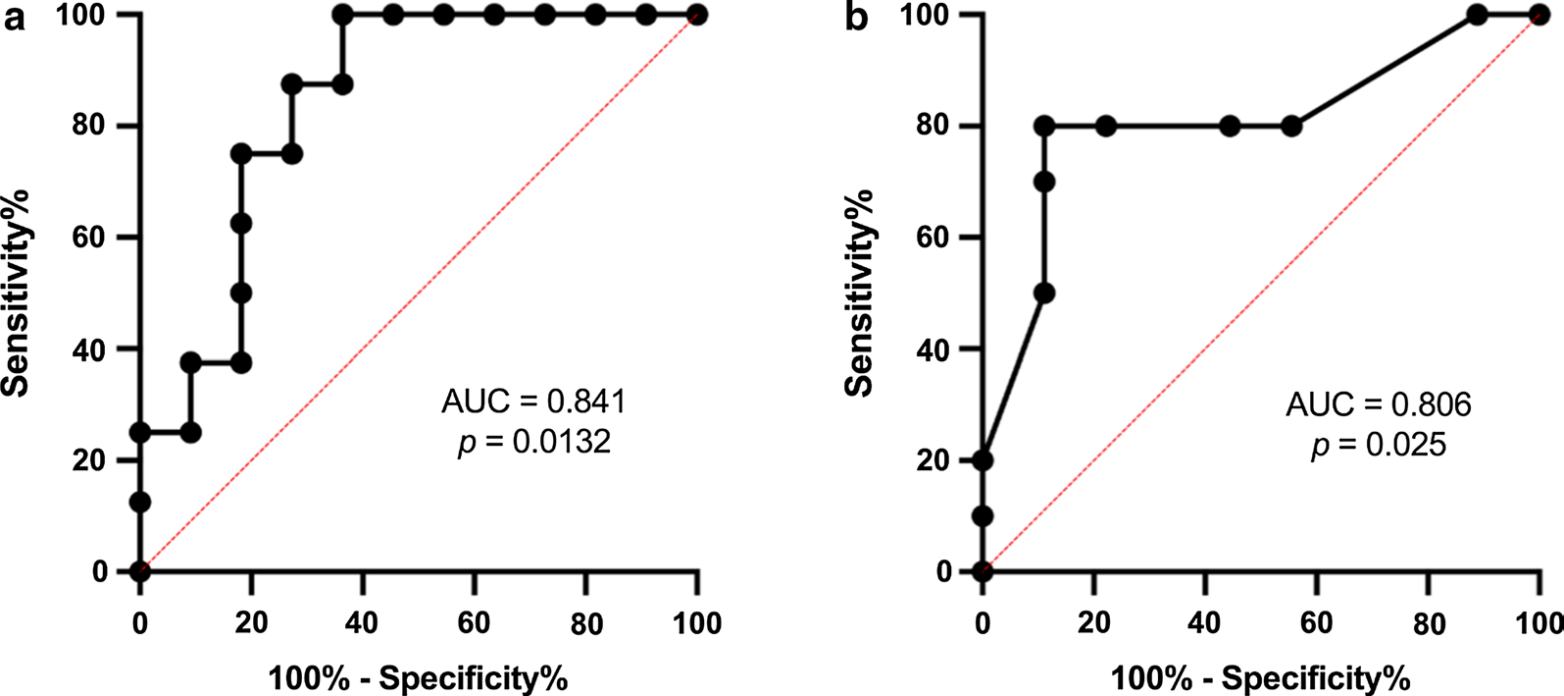
**a** Receiver Operating Characteristic Curve (ROC) for miR-126-5p in type 1 diabetes and healthy controls; **b** ROC curve of HbA1c indicating upregulation of miR-126-5p expression

Receiver operating characteristic curve (ROC) analyses showed that miR-126-5p was able to distinguish between T1DM and HC (AUC = 0.841, *p* = 0.0132) with a sensitivity of 87.5% and 72.73% specificity (Fig. 3a). In addition significant upregulation of miR-126-5p (AUC = 0.806, *p* = 0.025) with sensitivity of 80% and 89% specificity defined subclinical CVD at HbA1c > 38.25 mmol/mol (5.6%), *p* = 0.025 (Fig. 3b).

### Ingenuity pathway analysis (IPA) analysis

**Fig. 4:**
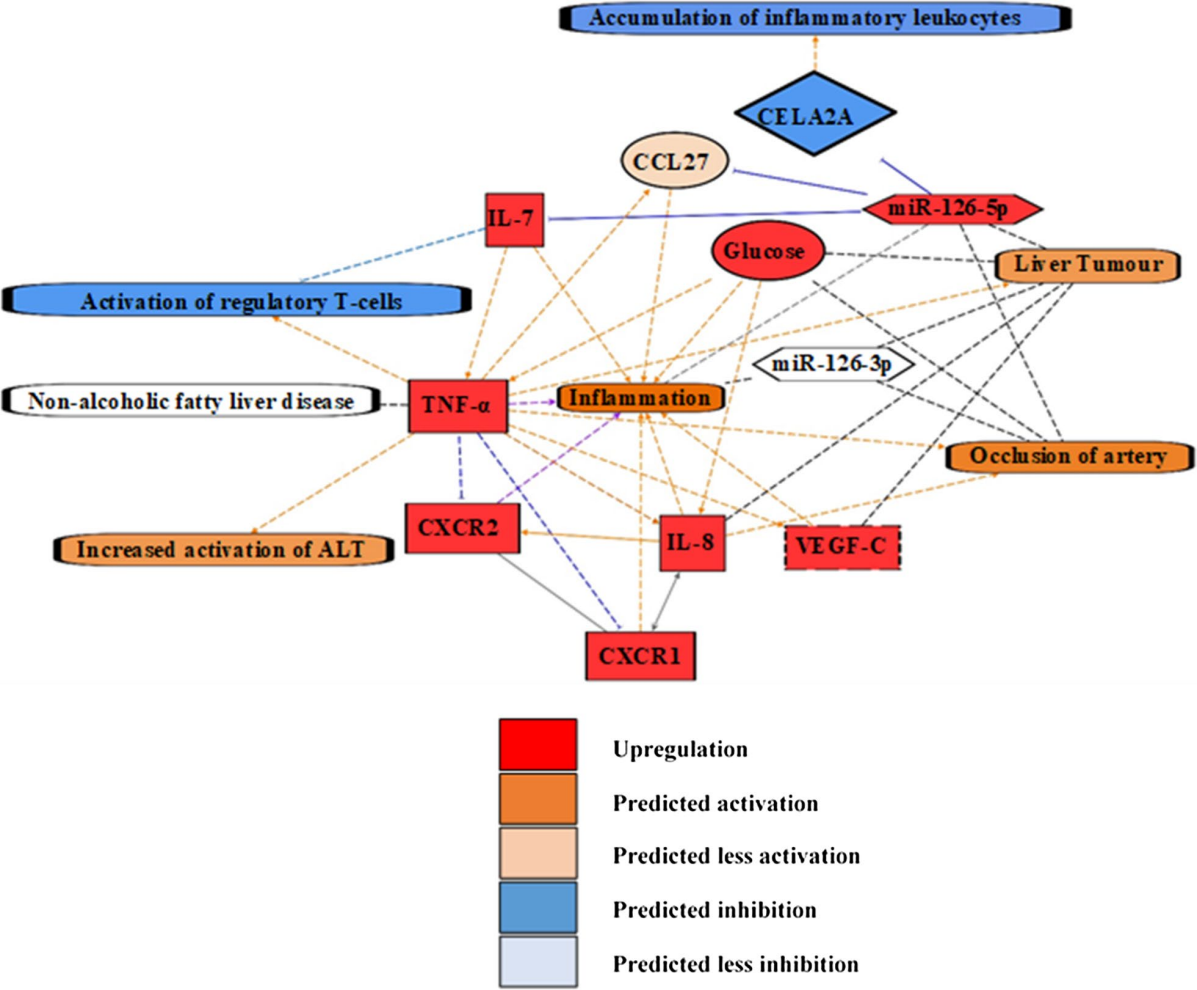
Ingenuity Pathway Analysis (IPA), prediction network of the T1DM conditions presented in this study with the upregulation (red) of miR-126-5p, IL-7, TNF-α, VEGF-C, CXCR1, CXCR2 and glucose. Orange signifies predicted activation of a molecule or biological function. Paler orange shades signify a prediction of less activation. Blue signifies inhibition of a molecule or biological function. White represents a molecule or biological function is unchanged. Orange lines represent stimulation, blues lines represent inhibition, the black lines represent a correlation, grey lines represent reciprocal stimulation and inhibition, and purple lines represents simultaneous stimulation and inhibition. Solid un-interrupted lines represent a direct action and dashed interrupted lines represent an indirect action. Alanine aminotransferase (ALT)

IPA software predicted from previously published data that miR-126-5p was anti-inflammatory through the inhibition of chemokine C–C motif ligand 27 (CCL27), chymotrypsin-like elastase 2A (CELA2A) and IL-7, whilst miR-126-3p had no anti-inflammatory effect. Simultaneously IPA predicted IL-7 to be the most upstream cytokine target (Fig. 4).

## Discussion

In this study, patients with T1DM without known CVD were considered as a model of subclinical cardiovascular disease. Our previous research documented asymptomatic T1DM patients to have features of early CVD [6, 8, 9]. Patients with T1DM were shown to display a significant endothelial dysfunction, inflammatory state, reduced circulating endothelial progenitor cells (cEPCs), pro-angiogenic cells (PACs) and colony forming units (Hills) [6, 8]. All of those vascular health indicators have been noted to characterize subclinical CVD in other patient groups [10, 11]. In the current study, we have demonstrated in T1DM in the presence of inflammatory state, an unexpected to our hypothesis, an upregulation of miR-126-5p in PBMCs but not in plasma whilst expression of miR-126-3p in both plasma and PBMCs was unchanged.

### T1DM is an inflammatory disease

In T1DM, the elevation of pro-inflammatory cytokines such as TNF- α, VEGF-2. IL-8 and homeostatic cytokine IL-7 is now well established [7, 9, 12].

**Fig. 5:**
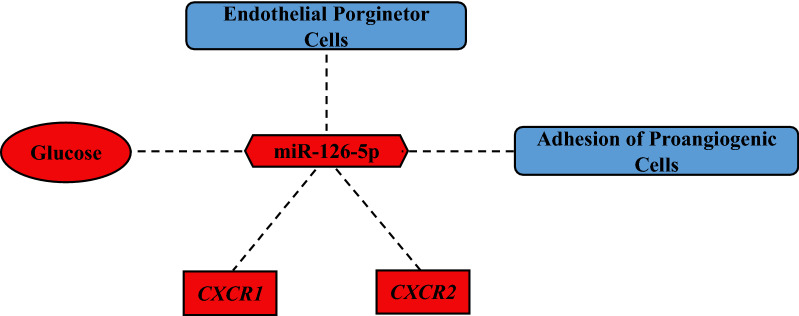
A graphical representation of the interplay of miR-126-5p with vascular health and inflammation in T1DM. Red represents upregulation of a molecule or function whilst blue represents suppression of a molecule or function. Dashed lines represent indirect causation

In this study we modelled an elevation of miR-126 in relation to established by us earlier, an inflammatory state in T1DM (Fig. 5). There are several cytokines identified by us to be upregulated. IL-7 is a homeostatic cytokine important in promoting T-lymphocyte survival [13]. IL-7 is demonstrated to generate CD8^+^ autoreactive stem memory T cells, which cause chronic autoimmune destruction of pancreatic beta-cells [13]. Moreover, IL-7 accelerated T1DM pathogenesis in mice through the expansion of autoreactive CD4^+^ and CD8^+^ T cells [14]. These studies align with and explain our clinical finding of increased levels of IL-7 in T1DM and implicate IL-7 in T1DM pathogenesis.

IL-8 is a pro-inflammatory cytokine with roles in chemo-attraction and neutrophil activation, which are mediated through differential activation of CXCR1 and CXCR2 receptors [15]. In line with our study elevated IL-8 has previously been demonstrated in T1DM patients [12]. In mouse models of T1DM inhibition of the IL-8 receptors CXCR1 and CXCR2, reduced insulitis whilst both preventing and reversing T1DM [16]. In our recently published study, we reported elevated *CXCR1* and *CXCR2* mRNA in T1DM [7].

Overall, other studies along with our own work indicate elevated IL-8 and IL-8 receptor (CXCR1/2) levels to be important for the pathogenic disease mechanism of T1DM [12, 15, 16].

The first and most established pro-inflammatory cytokine TNF-α was also raised in our subjects [17]. Previously our group had established elevated TNF-α in physically-fit males with T1DM keeping with our current study and supportive of chronic inflammation in T1DM [9]. Elevated TNF-α has been demonstrated to be pro-inflammatory causing the destruction of pancreatic beta-cells, indicating high TNF-α as a pathogenic mechanism for T1DM [18]. Interestingly, TNF-α activating treatments have been shown to be anti-inflammatory through selectively destroying autoreactive T lymphocytes in T1DM patients and mice models [19, 20] and furthermore, causing the expansion of anti-inflammatory T regulatory cells [21]. The opposing actions of TNF-α, highlights a counter-regulatory mechanism for the maintenance of pancreatic beta-cell numbers. These conflicting mechanisms were partly explained by Christen et al. [19], using T1DM mice models they demonstrated elevated levels of TNF-α is essential for early T1DM pathogenesis whereas, later in pathogenesis elevated TNF-α abrogated the autoimmune condition. We propose that early in T1DM pathogenesis TNF-α destroy beta-cells, however later in the pathogenesis, beta-cells are depleted therefore TNF-α may preferentially cause destruction of the autoreactive T lymphocytes and ameliorate T1DM. Overall, elevated TNF-α is indicated to be important for the pathogenesis of T1DM which is consistent with the elevated levels of TNF-α in our middle-aged T1DM cohort. However, it suggests that later in the T1DM disease progression elevated TNF-α level switches from a pathogenic function to a more therapeutic role, which supports the proposal for activating TNF-α for T1DM treatment [19]. However, evidence of this phenomenon in animal models and humans is minimal [19, 20] whilst anti-TNF-α treatments are already well established for the treatment of chronic inflammatory diseases in humans. Therefore, we propose anti-TNF-α treatments maybe more beneficial for T1DM treatment, whilst TNF-α-activating treatments are subject to further research.

We have previously reported our finding of an increase in expression of the growth factor VEGF-C in T1DM [7]. VEGF-C is involved in the formation of lymphatic vessels and angiogenesis [22, 23]. VEGF-C was observed to increase inflammation [24] whilst lymphangiogenesis, a function of VEGF-C, is observed to be increased in chronic inflammatory diseases including inflammatory bowel diseases [25]. Therefore, we postulate elevated VEGF-C is causal to the chronic inflammatory state in T1DM. VEGF-C is downstream from the more regulatory cytokines IL-7 and TNF-α, which when upregulated increase VEGF-C expression and drive inflammation as a secondary effect, as displayed by our IPA analysis (Fig. 4). Overall, we hypothesise that elevated VEGF-C is a contributing factor to the inflammatory state in T1DM.

### MiR-126-3p/-5p expression in T1DM

The strength of our study lies in that we measured simultaneously both miR-126-3p and miR-126-5p in the same tissues and this allows us to make a comparison and provide an insight into the differing expression of the two miR-126 isoforms in T1DM.

Previously miR-126-3p/-5p have been demonstrated to promote vascular integrity and angiogenesis through the use of miR-126-3p/-5p knockdown zebrafish models [4]. MiR-126-3p delivered in apoptotic bodies was demonstrated to prevent atherosclerosis in atherosclerotic mice, by conferring plaque stability [26]. However further research suggest that these two miRNAs have different function. In double miR-126-3p/-5p knockout atherosclerotic mouse model, re-introducing miR-126-5p expression alone increased endothelial cell proliferation and ameliorated atherosclerosis via reduction of atherosclerotic lesion formation [5]. In contrast miR-126-3p re-introduced into the double knockout mouse failed to increase endothelial cell proliferation and improve atherosclerosis. These studies suggest miR-126-5p has more importance in angiogenesis and cardio-protection than miR-126-3p.

Aligned with our current work Takahashi et al. similarly demonstrated upregulation of miR-126-5p in T1DM PBMCs but also demonstrated upregulation of miR-126-3p in contrast to our finding on miR-126-3p [27]. The discrepancy in the latter finding may relate to Takahashi et al. studying a younger cohort of T1DM patients (23.5 ± 3.9 years) compared to our present study (47.4 ± 14.5 years) or the methodology used [27]. Previously, in T1DM miR-126-3p was observed to be downregulated in the urine of paediatric patients but in plasma they were unchanged as in our previous and current studies [28, 29]. In T1DM patients of a similar age to our current study, a downregulation has been demonstrated in miR-126-3p in both endothelial progenitor cells and serum [30, 31]. We propose that age, tissue assayed, and methodology may be confounding factors that influence miR-126-3p/-5p expressions. MiR-126-3p was downregulated in plasma in type 2 diabetes [32] and miR-126-5p was downregulated in myocardial tissue in ischemic reperfusion injury patients [33]. Elucidating the tissue- and disease-specific expression of miR-126 isoforms is useful for the development not only of biomarkers but also for novel miRNA-based therapeutics. PBMC are likely to take part in the inflammatory response thus the elevation of miR-126-5p in those cells may be considered as pro-angiogenic and anti-inflammatory. ROC analysis provided evidence that miR-126-5p can discriminate with high significance between disease state (subclinical CVD) and health and this occurred at the level of HbA1c > 38.25 (5.6%). We thus postulate that miR-126-5p is a biomarker for early subclinical CVD as the difference in the expression between T1DM and HC could be attributed to the onset of hyperglycaemia.

### Correlation of miR-126-5p with hyperglycaemia and inflammation

We found direct correlation between miR-126-5p and HbA1c and IL-8 receptor CXCR1/2 mRNA (Fig. 2 a-c). It is well established that the causality cannot be established from any correlation analysis. However our data suggest the greater hyperglycaemia or inflammatory state the greater miR-126-5p expression. We thus postulate, given animal research, that miR-126-5p upregulation may be compensatory.

### Correlation of miR-126-5p with vascular health

This study shows inverse correlations between miR-126-5p and circulating endothelial progenitor cells (cEPCs) defined as CD45dimCD34^+^CD133^+^cells. EPCs are known to represent vascular health/ repair and have been established as predictive marker for future vascular events [6, 8, 10, 34]. The finding of better vascular health associated with the lower miR-126-5p expression, supported our interpretation of lesser requirement for compensation from proangiogenic miR-126-5p. Similar correlation was noted in our study between miR-126-5p and fibronectin adhesion assay. FAA represents cell adhesion crucial for cell regeneration, motility and angiogenesis [8, 35]. Thus the better cell adhesion, the lesser requirement for miR-126.-5p compensation. It is interesting to note that two independent measures of vascular health (cEPCs and FFA) in our study showed the same relation to proangiogenic miR 126-5p, thus internally validated our results. The summary of our data is illustrated in Fig. 5.

In concordance with our work, a recent study highlighted elevated miR-126-5p in established CVD in patients (acute myocardial infarction) whilst receiver operating characteristic analyses indicated miR-126-5p to show considerable diagnostic capability for diagnosis of acute myocardial infarction [36]. Our interpretation of Xue et al. research: given animal knockout experiments showing clear proangiogenic function of miR-126-5p in reducing atherosclerosis, we postulate that compensatory response is activated in patients with myocardial infarction too and this overexpression defined the diagnosis of acute myocardial infarction.

### Ingenuity Pathway Analysis (IPA) predicts miR-126-5p as anti-inflammatory under our T1DM conditions

In order to verify the interpretation of our results we run independent IPA. IPA included miR-126-5p, IL-7, IL-8, TNF-α, VEGF-C, CXCR1 and CXCR2 and glucose, all upregulated in this study to replicate T1DM conditions. IPA generated independently indirect or direct causations between targets and made predictions of inhibitory or stimulatory actions based on previously published evidence (Fig. 4).

IPA indicated miR-126-5p to inhibit CELA2A which reduced accumulation of inflammatory leukocytes (Fig. 4). Furthermore miR-126-5p was predicted to inhibit the chemokine C–C motif ligand 27 (CCL27) and cytokine IL-7, which limit the pro-inflammatory actions of both CCL27 and IL-7 (Fig. 4). Meanwhile elevated IL-7, IL-8, TNF-α, glucose, VEGF-C, CXCR1 and CXCR2 were predicted to enhance inflammation (Fig. 4). Furthermore, unsurprisingly IPA confirmed that elevated glucose levels increased TNF-α and IL-8 expression (Fig. 4). Notably TNF-α and CXCR2 were predicted to have a dual role with pro-inflammatory actions and anti-inflammatory actions (Fig. 4). Anti-inflammatory actions of TNF-α included the activation of T regulatory lymphocytes and suppressing inflammation, which is concordant with the dual role of TNF-α in T1DM (Fig. 4) [18–21]. However, IL-7 was predicted to inhibit the activation of anti-inflammatory regulatory T-cells, with a dominant action over TNF-α resulting in the inhibition of the anti-inflammatory regulatory T-cells in these T1DM conditions (Fig. 4). Interestingly the elevated levels of IL-7 were predicted to indirectly activate TNF-α, in turn TNF-α indirectly activated VEGF-C, IL-8 and CCL27 (Fig. 4). This demonstrated IL-7 to be the most upstream cytokine, which indirectly regulated the expression of other cytokines. Overall, based on previous studies, IPA indicated elevated miR-126-5p to represent an anti-inflammatory response in T1DM, which limits the effect of cytokines. Furthermore, IPA indicated IL-7 as the most upstream cytokine and to be the most suitable target, to manage the inflammation in T1DM.

Additionally, IPA predicted the elevated TNF-α and IL-8 to be causal to atherosclerotic disease (Fig. 4). Negative correlations of miR-126-3p/-5p with atherosclerosis, and a positive correlation of glucose with atherosclerosis have been predicted in IPA (Fig. 4).

Elevated TNF-α was predicted by IPA to activate ALT levels and be causal to liver tumours. Furthermore TNF-α expression was positively correlated with non-alcoholic fatty liver disease (NAFLD), confirming NAFLD as an inflammatory disease (Fig. 4). Positive correlations of miR-126-3p, miR-126-5p, VEGF-C and IL-8 with liver tumours have been predicted, whilst a negative correlation of glucose with liver tumours exists (Fig. 4).

Unsurprisingly IPA demonstrated CXCR1 and CXCR2 to be receptors for IL-8, whilst CXCR1 and IL-8 share a relationship of reciprocal activation and inhibition (Fig. 4). CXCR1 and CXCR2 are also predicted to bind each other with protein–protein interactions (Fig. 4).

MiR-126-3p in the IPA prediction network was positively correlated with liver cancer and negatively correlated inflammation and atherosclerotic disease (Fig. 4). The absence of direct or indirect interaction of miR-126-3p with inflammation, CVD or inflammatory liver disease was in concordance with our finding of no change in miR-126-3p expression in T1DM.

Thus IPA analysis by using previously published evidence highlighted targets for therapies for the management of CVD and inflammation.

### Translational value of our findings

Given the mechanistic *in-vivo* studies published previously demonstrating miR-126-5p as cardioprotective in animal models we postulate miR-126-5p upregulation in T1DM is compensatory [4, 5, 37]. This is consistent with IPA pathways analysis.

Importantly, in the long-term further studies are necessary to confirm our findings in a larger cohort of T1DM patients before pro-miR-126-5p therapies can be developed. However, in the short-term, a possible solution is to investigate further pro-miR-126-5p in animal models of T1DM to gain insight into their mechanism of action.

Current methodologies for anti-miRNA-based therapies entail anti-miRNAs linked with anti-sense oligonucleotides which can reduce miRNA concentrations [38]. Anti-miRNA therapies have demonstrated atheroprotective effects in atherosclerotic mice by preventing endothelial dysfunction [39]. Overall indicating pro-miR-126-5p therapies as potential treatment strategies for the management of T1DM and the associated CVD morbidities.

As miR therapies have multiple targets, focused therapy will not be easy. Thus it is of advantage to know of more focused approach to human diseases using monoclonal antibodies against certain cytokines, already used in clinical practice.

### IL-7 therapy

To date no clinical trial data on therapies targeting IL-7 for chronic inflammatory diseases has been published. However, in animal models a blockade of the IL-7 receptor has shown efficacy in ameliorating chronic inflammatory disease in rodent models of T1DM [14] and multiple sclerosis [40]. Blocking of IL-7 with monoclonal antibodies also produced better control of skin inflammation in non-human primates through the negative regulation of memory T cell responses [41]. These findings in corroboration with our study indicate targeting of the IL-7 to be a potential therapeutic target for the management of T-cell mediated chronic inflammatory disease such as T1DM in humans.

### IL-8 therapy

No clinical trial data on IL-8 based therapy is currently available. Pharmacological inhibition of IL-8 receptors was observed to reverse T1DM pathogenesis and reduce pancreatic inflammation [16]. Furthermore the concentration of IL-8 was observed to be elevated in mice following myocardial infarction whilst treatment with Pterostilbene, a natural anti-inflammatory phytoalexin, decreased IL-8 levels and subsequently led to smaller myocardial infarct size and reduced cardiomyocyte apoptosis [42]. Elevated IL-8 has been found in patients with acute myocardial infarction, suggesting IL-8 to be involved in myocardial injury [43]. Overall, our data and other’s data suggest IL-8 based therapy may be useful for the management of CVD and in particular in T1DM.

### TNF-α therapy

Current TNF-inhibitors approved for treatment of chronic inflammatory diseases include full-length bivalent IgG monoclonal antibodies: adalimumab, golimumab and infliximab, a soluble receptor etanercept and a PEGylated Fab fragment of a monoclonal antibody certolizumab. Patients with inflammatory arthritis including rheumatoid arthritis, ankylosing spondylitis and psoriatic arthritis are at a higher risk of CVD. Anti-TNF therapy administered to patients with inflammatory arthritis experienced reductions in myocardial inflammation quantified by reductions in myocardial oedema and extracellular volume of the heart, whilst improvements in C-reactive Protein and Erythrocyte Sedimentation Rate were observed [43]. Moreover Anti-TNF causes improvements in cardiovascular function including improvements in systolic aortic strain, diastolic aortic strain, aortic stiffness and myocardial oedema [44] and reduction in the incidence of major cardiovascular events [45]. This confirms the promise for anti-TNF-α therapy in T1DM for the management of CVD.

### VEGF-C therapy

VEGF-C based therapies are currently not in clinical trials. Our current work and other implicate VEGF-C to promote inflammation, however surprisingly the activation of VEGF-C or the VEGF-C receptor has shown to be anti-inflammatory [25, 45]. Adenoviral induction of VEGF-C in inflammatory bowel disease mice models reduced gut inflammation by increasing lymphangiogenesis and improving lymphatic function [25]. Furthermore, activation of the VEGF-C receptor, VEGFR3, inhibited chronic skin inflammation in mice via enhancing lymphangiogenesis [46]. We propose elevated VEGF-C to be important initially to stimulate lymphangiogenesis and lymphatic function to allow the pathogenesis of chronic inflammatory diseases, however when a certain threshold of VEGF-C is surpassed, through activation of VEGF-C or the VEGF-C receptor, the greater extent of lymphangiogenesis and lymphatic function is such that it counteracts the initially pathogenic pro-inflammatory effects of VEGF-C. These studies highlight the potential of enhancing VEGF-C for the treatment of chronic inflammatory diseases and for other inflammatory conditions including T1DM.

## Conclusions

T1DM is an inflammatory disease characterised by elevated pro-inflammatory cytokines with elevated *CXCR1* and *CXCR2* mRNA cytokine receptor. MiR-126-5p upregulation in T1DM may be a compensatory response, augmented by hyperglycaemia and inflammation. IL-7 is the most upstream cytokine whilst being the most suitable target, to manage the inflammation in T1DM/CVD. Thus, there is a plethora of potential strategies for limiting CVD complications of T1DM: either by targeting glucose, IL-7, CXCR1/2 or the novel application of pro-miR-126-5p therapies. Further studies on a larger cohort of patients are required to confirm our current findings.

## Data Availability

The datasets analysed during the current study is available from the corresponding author on reasonable request.

## Abbreviations

CCL27: Chemokine C–C motif ligand 27
CELA2A: Chyotrypsin-like elastase 2A
CVD: Cardiovascular disease
CXCR1/2: Chemokine C-X-C receptor 1/2
HC: Healthy control
IPA: Ingenuity pathway analysis
miRNA: MicroRNA
NAFLD: Non-alcoholic fatty liver disease
PBMC: Peripheral blood mononuclear cell
qRT-PCR: Quantitative real-time PCR
RIN: RNA integrity number
T1DM: Type 1 diabetes
VEGF-C: Vascular endothelial growth factor-C
